# Clinical application of CT and CT-guided percutaneous transthoracic needle biopsy in patients with indeterminate pulmonary nodules*

**DOI:** 10.1590/S1806-37132014000400005

**Published:** 2014

**Authors:** Luciana Vargas Cardoso, Arthur Soares Souza

**Affiliations:** São José do Rio Preto Hospital de Base, São José do Rio Preto School of Medicine, São José do Rio Preto, Brazil; Graduate Program at São José do Rio Preto School of Medicine; and Administrative Director. Rio Preto-Ultra-X Radiological Diagnosis Institute, São José do Rio Preto, Brazil

**Keywords:** Solitary pulmonary nodule, Tomography, Image-guided biopsy

## Abstract

**OBJECTIVE:**

To investigate the clinical application of CT and CT-guided percutaneous transthoracic needle biopsy (CT-PTNB) in patients with indeterminate pulmonary nodules (IPNs).

**METHODS:**

We retrospectively studied 113 patients with PNs undergoing CT and CT-PTNB. Variables such as gender, age at diagnosis, smoking status, CT findings, and CT-PTNB techniques were analyzed. Data analysis was performed with the Student's t-test for independent samples the chi-square test, and normal approximation test for comparison of two proportions.

**RESULTS:**

Of the 113 patients studied, 68 (60.2%) were male and 78 (69%) were smokers. The diameter of malignant lesions ranged from 2.6 cm to 10.0 cm. Most of the IPNs (85%) were located in the peripheral region. The biopsied IPNs were found to be malignant in 88 patients (77.8%) and benign in 25 (22.2%). Adenocarcinoma was the most common malignant tumor, affecting older patients. The IPN diameter was significantly greater in patients with malignant PNs than in those with benign IPNs (p < 0.001). Having regular contour correlated significantly with an IPN being benign (p = 0.022), whereas spiculated IPNs and bosselated IPNs were more often malignant (in 50.7% and 28.7%, respectively). Homogeneous attenuation and necrosis were more common in patients with malignant lesions (51.9% and 26.9%, respectively)

**CONCLUSIONS:**

In our sample, CT and CT-PTNB were useful in distinguishing between malignant and benign IPNs. Advanced age and smoking were significantly associated with malignancy. Certain CT findings related to IPNs (larger diameter, spiculated borders, homogeneous attenuation, and necrosis) were associated with malignancy.

## Introduction

Some of the greatest challenges in the fields of thoracic surgery and radiology are related to the evaluation and management of pulmonary nodules.^(^ 1 ^)^ A pulmonary nodule is defined as a well-demarcated, round focal opacity visible on chest X-rays or CT scans and surrounded by normal lung tissue, being up to 3 cm in diameter; pulmonary nodules larger than 3 cm are designated masses.^(^ 2 ^)^

It is extremely important to investigate pulmonary nodules because they constitute the most common manifestation of lung cancer, being a common finding on chest CT scans.^(^ 3 ^)^ In the USA, approximately 150,000 pulmonary nodules are detected each year.^(^ 3 ^,^ 4 ^)^ Of all pulmonary nodules seen on imaging, 60-70% are benign and 30-40% are malignant.^(^ 4 ^)^

A pulmonary nodule requires careful patient evaluation, including clinical history taking, physical examination, evaluation of risk factors for malignancy, and diagnostic imaging.^(^ 3 ^,^ 5 ^)^ Diagnostic imaging methods for distinguishing between benign and malignant pulmonary nodules include X-rays, CT, magnetic resonance imaging, positron emission tomography/CT, and CT-guided percutaneous transthoracic needle biopsy (CT-PTNB). 

Helical CT is critical in distinguishing between benign and malignant nodules, providing data on size, margins, and the presence of internal calcification. In addition, helical CT images can show nodular enhancement after intravenous contrast administration. Furthermore, helical CT allows greater accuracy in obtaining biopsy specimens.^(^ 6 ^,^ 7 ^)^ Size, location, margins, contents, contrast enhancement, and doubling time are some of the nodule features that can be seen on CT scans of patients with pulmonary nodules, principally on those of those who are male, are over 50 years of age, are smokers, and have a family history of cancer or pulmonary fibrosis. 

CT-PTNB has been widely used in the investigation of pulmonary nodules and masses. Samples can be collected by fine-needle aspiration biopsy (FNAB) or thick-needle aspiration biopsy, the latter being known as core biopsy.^(^ 8 ^)^ Core biopsy has greatly contributed to a specific and early diagnosis of malignancy in patients with pulmonary nodules, reducing morbidity and mortality rates.^(^ 8 ^)^

The differential diagnosis of pulmonary nodules includes various diseases and tumors. Benign nodules include hamartomas, granulomas, and intrapulmonary lymph nodes.^(^ 4 ^)^ Infectious granulomas account for 90% of all benign nodules and are most commonly caused by tuberculosis, histoplasmosis, and coccidioidomycosis.^(^ 4 ^)^ The most common malignant tumors include adenocarcinoma and epidermoid carcinoma.^(^ 4 ^)^

Several CT criteria have been used in order to distinguish between benign and malignant nodules. Poorly demarcated nodules, absence of calcification (central, laminated, diffuse, or "popcorn" calcification) or fat in the lesion, doubling time ranging from one month to one year approximately, and nodular enhancement greater than 15 HU after intravenous contrast administration in patients past the fourth decade of life are suggestive of malignancy.^(^ 7 ^,^ 9 ^,^ 10 ^)^ Small, well-demarcated nodules with concentric or "popcorn" calcification in young patients are suggestive of benign lesions.^(^ 10 ^)^ The absence of lesion growth for at least two years is also suggestive of benignity.^(^ 11 ^)^

The present study is warranted because we found no studies examining the clinical application of CT and CT-PTNB in patients with pulmonary nodules in Brazil. From a clinical standpoint, early detection and CT-PTNB of malignant lesions can, in some cases, avoid invasive procedures, such as bronchoscopic biopsy, video-assisted thoracoscopic surgery, and even unnecessary surgery. They can also avoid the progression of lung cancer to advanced stages, enhancing patient quality and quantity of life.^(^ 3 ^,^ 12 ^)^

The objective of the present study was to investigate the clinical application of CT and CT-PTNB in patients with indeterminate pulmonary nodules, demographic characteristics, CT features, and CT-PTNB findings, as well as their correlation with the histopathological diagnosis, being taken into consideration. 

## Methods

Of a total of 132 patients with pulmonary nodules and masses studied between June of 2006 and May of 2007, 113 (85.6%) were retrospectively investigated (regardless of gender, age, or race), having undergone helical CT and CT-PTNB. The procedures were performed in the Department of Radiology of the São José do Rio Preto School of Medicine São José do Rio Preto *Hospital de Base*, located in the city of São José do Rio Preto, Brazil. The study was approved by the local research ethics committee (Protocol no. 3682/2006). 

We excluded 19 patients whose histopathological reports showed unsatisfactory or inconclusive results because of insufficient material. 

The following data were collected from patient charts: gender; age at diagnosis; smoking status; CT findings, such as diameter (≤ 3 cm for nodules and > 3 cm for masses),^(^ 2 ^)^ location (central or peripheral), lesion margins (regular, irregular, spiculated, or bosselated), and intralesional changes (homogeneous attenuation, necrosis, cavitation, calcification, and air bronchogram); CT-PTNB technique used (FNAB, core biopsy, or both); and complications. 

The CT findings were independently evaluated by two radiologists who were blinded to the histopathological findings. 

All CT examinations were performed with a Tomoscan^(r)^ SR 4000 CT scanner (Phillips Medical Systems, Eindhoven, the Netherlands). Ten-millimeter CT scans of the chest were taken from the lung apices to the bases during inhalation, a high-resolution filter being used for image reconstruction. Subsequently, helical CT scans were taken before and after intravenous injection of a nonionic contrast medium, the following parameters being used: slice thickness, 10 mm; pitch (ratio between table movement per rotation and slice thickness), 2 cm; 120 kVp; and 150 mA. 

Patients undergoing CT-PTNB were evaluated for general health, level of consciousness, pulmonary functional reserve, and coagulation parameters. All patients were informed of the complications of CT-PTNB and were instructed to hold their breath during the examination. The procedure was performed without intravenous contrast, during single breath-hold maneuvers performed during inhalation, with patients in the supine or prone position in order to allow direct access to the lesion. 

The CT-PTNB protocol used in the radiology department of the institution is as follows: slice thickness, 5-10 mm; pitch, 2 cm; 120 kVp; and 150 mA. The goals are to locate the lesion, determine the site at which the needle should be introduced, and measure needle distance and angle. Sterilization of the puncture site was achieved with povidone-iodine, a sterile field being created with surgical drapes. Patients then received 10 mL of local anesthetic (2% lidocaine). A small incision was made with a scalpel (no. 14 blade), the needle being introduced into subcutaneous tissue through the incision. CT scans were taken in order to locate the tip of the needle, which was attached to a Bard Magnum^(r)^ automatic pistol (Manan Medical Products, Northbrook, IL, USA). 

CT-PTNB was performed by FNAB, core biopsy, or both. Needles ranging from 18 G to 20 G were used for core biopsy, and needles ranging from 22 G to 25 G were used for FNAB. After having undergone biopsy, patients were monitored for 2-3 h, CT scans being taken in order to detect complications. 

For data analysis, descriptive and inferential statistics were used. For comparison of means, we used the Student's t-test for independent samples (distribution of benign and malignant lesions by age and pulmonary nodule diameter), the chi-square test (distribution of benign and malignant lesions by gender, lesion location, smoking status, and CT-PTNB technique), and the normal approximation test for comparison of two proportions (distribution of benign and malignant lesions by lesion margins and intralesional changes).^(^ 13 ^)^ The level of significance was set at p < 0.05. All analyses were performed with Minitab software, version 15 (Minitab Inc., State College, PA, USA).^(^ 14 ^)^

## Results

Of the 113 patients studied, 68 (60.2%) were male and 45 (39.8%) were female. The mean age was 59.3 ± 12.6 years, and the median age was 61 years (range, 12-82 years). Of the 113 patients studied, 78 (69%) were smokers and 35 (31%) were nonsmokers. Of the 78 smokers, 48 (61.5%) were male and 30 (38.5%) were female. 

**Figure 1:**
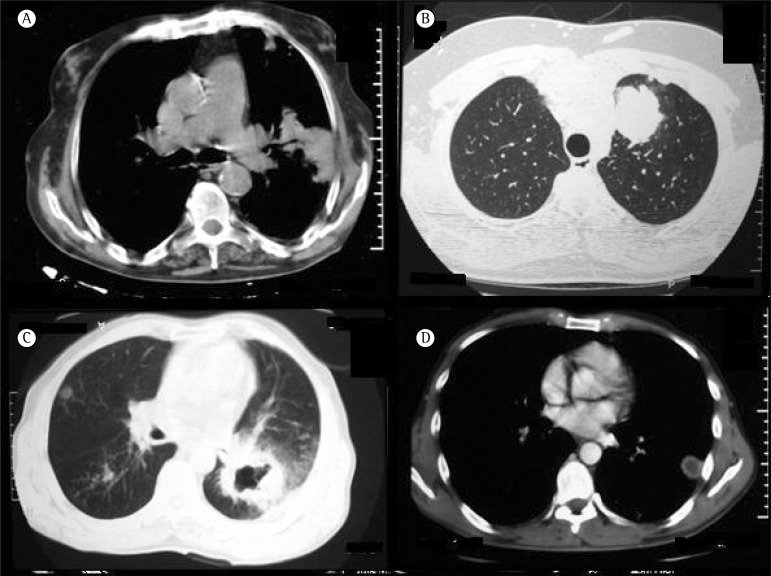
Helical CT scans showing an irregular lung mass (in A; male patient, 77 years old), a bosselated lung mass (in B; male patient, 30 years old), a spiculated lung mass (in C; male patient, 64 years old), and a regular lung mass (in D; male patient, 36 years old)

**Figure 2:**
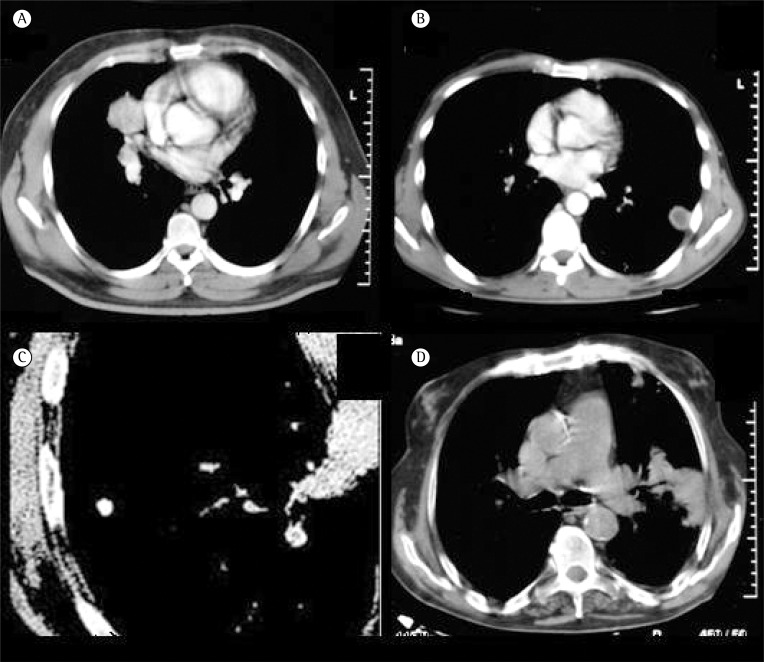
Helical CT scans showing intralesional changes, including homogeneous attenuation (in A; male patient, 49 years old), necrosis (in B; male patient, 36 years old), calcification (in C; male patient, 56 years old), and air bronchogram (in D; male patient, 77 years old).

The diameter of benign lung lesions ranged from 1.8 cm to 6.5 cm, and that of malignant lung lesions ranged from 2.6 cm to 10.0 cm. The difference between benign and malignant nodules/masses was statistically significant (p = 0.003), malignant nodules and masses having predominated (23.0% and 54.8%, respectively). Most (85%) of the pulmonary nodules were located in the peripheral region, and 15% were located in the central region. There was a predominance of malignant tumors in the upper lobes, in 67 patients (76%). Of the 185 nodules found in the 113 patients studied, spiculated nodules were the most common (49.7%), followed by bosselated nodules (26.5%), irregular nodules (12.4%), and regular nodules (11.4%; Figure 1). The CT scans showed a total of 151 intralesional changes, the most common being homogeneous attenuation (42.4%), followed by necrosis (21.2%), cavitation (17.2%), calcification (11.2%), and air bronchogram (8.0%; Figure 2). 

FNAB was performed in 71 patients, core biopsy was performed in 81, and a combination of the two was performed in 39. Pneumothorax was the only complication of CT-PTNB, in 37 patients (32.7%). Histopathology revealed that the pulmonary nodules were malignant in 88 (77.8%) of the 113 patients and benign in 25 (22.2%). 

Adenocarcinoma was the most common malignant tumor (48.9%), affecting older patients (mean age, 65.6 ± 9.1 years). Malignant lesions ranged from 2.4 cm to 10.0 cm in diameter, whereas benign lesions ranged from 1.8 cm to 6.5 cm in diameter. 

Patients with malignant lesions were found to be older than those with benign lesions, the difference being significant (p = 0.034); there was also a significant difference between benign and malignant lesions in terms of their size (p < 0.001), malignant lesions being larger in diameter (Table 1). 

**Table 1:** Distribution of malignant and benign lesions in the study sample (N = 113), by patient age and pulmonary nodule diameter.

Variable	Diagnosis	n	Mean ± SD	Median (range)	p*
Age	Malignant	88	60.7 ± 12.1	63 (30-80)	0.034
	Benign	25	54.4 ± 12.9	60 (12-82)	
Diameter	Malignant	88	5.3 ± 1.9	5.0 (2.4-10.0)	< 0.001
	Benign	25	3.7 ± 1.3	4.0 (1.8-6.5)	

* Student's t-test for independent samples.

As can be seen in Table 2, neither age nor nodule location were significantly associated with the histopathological diagnosis (p = 0.067 and p = 0.264, respectively). The presence of regular margins was significantly associated with a pulmonary nodule being benign (p = 0.022). Spiculated pulmonary nodules and bosselated pulmonary nodules were more often malignant (in 50.7% and 28.7%, respectively). All intralesional changes were significantly associated with the histopathological diagnosis. Homogeneous attenuation and necrosis were more common in patients with malignant lesions (51.9% and 26.9%, respectively), whereas cavitation, calcification, and air bronchogram were more common in those with benign lesions (29.8%, 23.4%, and 17.0%, respectively). In the calculations related to the tests for comparison of proportions (Table 2), the CT findings of lesion margins and intralesional changes were analyzed on the basis of the assumption that a given patient might present with different types of lesion margins or intralesional changes. 

**Table 2:** Distribution of malignant and benign lesions in the study sample (N = 113), by gender and CT findings.a

Parameter		Diagnosis		Total	p
		Malignant	Benign		
Gender	Female	39 (44.3)	06 (24.0)	45 (39.8)	0.067*
	Male	49 (55.7)	19 (76.0)	68 (60.2)	
	Total	88 (100.0)	25 (100.0)	113 (100.0)	
Location	Central	15 (17.0)	02 (8.0)	17 (15.0)	0.264*
	Peripheral	73 (83.0)	23 (92.0)	96 (85.0)	
	Total	88 (100.0)	25 (100.0)	113 (100.0)	
Lesion margins	Regular	12 (8.0)	16 (45.7)	28 (15.1)	0.022**
	Spiculated	76 (50.7)	09 (25.7)	85 (45.9)	0.597**
	Bosselated	43 (28.7)	06 (17.1)	49 (26.5)	0.118**
	Irregular	19 (12.6)	4 (11.4)	23 (12.4)	0.837**
	Total	150 (100.0)	35 (100.0)	185 (100.0)	
Intralesional changes	Homogeneous attenuation	54 (51.9)	10 (21.3)	64 (42.4)	0.001**
	Necrosis	28 (26.9)	04 (8.5)	32 (21.2)	0.007**
	Cavitation	12 (11.5)	14 (29.8)	26 (17.2)	0.004**
	Calcification	06 (5.7)	11 (23.4)	17 (11.2)	0.003**
	Air bronchogram	04 (4.0)	08 (17.0)	12 (8.0)	0.015**
	Total	104 (100.0)	47 (100.0)	151 (100.0)	

a Values expressed as n (%)

* Chi-square test

** Normal approximation test for comparison of two proportions.

There was a significant association between the presence of malignant lesions and smoking (p = 0.002). Most of the patients in the study sample were smokers (n = 78). Of those, 76.1% had malignant lesions. 

There was no significant association between the histopathological diagnosis and the CT-PTNB technique employed (p = 0.778). The proportions of lesions that were diagnosed as malignant by core biopsy, FNAB, or a combination of the two were similar, i.e., 29.2%, 23.0%, and 25.6%, respectively. 

Table 3 shows the percentage distribution of malignant lesions by gender and CT findings. Malignant lesions were more common in male patients (55.7%). Adenocarcinoma was the most common malignant lesion in males and females (48.9%). Regarding location, peripheral lesions predominated (82.9%). Adenocarcinoma was the most common tumor in the peripheral region (56.2%). Regarding lesion margins, approximately half of all lesions were spiculated (50.7%). In patients with adenocarcinoma, the most common lesions were those with irregular margins (57.9%), those with spiculated margins (51.3%), and those with bosselated margins (44.2%). Homogeneous attenuation was the most common intralesional change (51.9%), followed by necrosis (26.9%). Homogeneous attenuation was most commonly found in patients with adenocarcinoma and in those with epidermoid carcinoma (38.9% and 24.1%, respectively). Cavitation was most common in cases of epidermoid carcinoma (66.7%). 

**Table 3:** Distribution of malignant lesions in the study sample (N = 113), by gender and CT findings.a

Parameter	Malignant lesion							
	ADC	EPC	HL	SCC	NHL	MT	Other	Total
Gender								
Female	15 (38.5)	11 (28.2)	5 (12.8)	0 (0.0)	2 (5.1)	1 (2.6)	5 (12.8)	39 (44.3)
Male	28 (57.1)	5 (10.2)	3 (6.1)	6 (12.2)	2 (4.1)	3 (6.1)	2 (4.1)	49 (55.7)
Total								88 (100)
Location								
Central	2 (13.3)	0 (0.0)	8 (53.3)	2 (13.3)	1 (6.7)	0 (0.0)	2 (13.3)	15 (17.0)
Peripheral	41 (56.2)	16 (21.9)	0 (0.0)	4 (5.6)	3 (4.1)	4 (5.6)	5 (6.8)	73 (83.0)
Total								88 (100)
Lesion margins								
Regular	4 (33.3)	4 (33.3)	1 (8.3)	0 (0.0)	0 (0.0)	1 (8.3)	2 (16.7)	12 (8.0)
Spiculated	39 (51.3)	12 (15.8)	7 (9.2)	6 (7.9)	4 (5.3)	3 (3.9)	5 (6.6)	76 (50.7)
Bosselated	19 (44.2)	6 (14.0)	7 (16.3)	3 (7.0)	1 (2.3)	3 (7.0)	4 (9.3)	43 (28.7)
Irregular	11 (57.9)	3 (15.8)	0 (0.0)	3 (15.8)	0 (0.0)	1 (5.3)	1 (5.3)	19 (12.6)
Total								150 (100)
Intralesional changes								
Homogeneous attenuation	21 (38.9)	13 (24.1)	7 (13.0)	3 (5.6)	1 (1.9)	4 (7.4)	5 (9.3)	54 (51.9)
Necrosis	17 (60.7)	3 (10.7)	1 (3.6)	2 (7.1)	3 (10.7)	0 (0.0)	2 (7.1)	28 (26.9)
Cavitation	1 (8.3)	8 (66.7)	0 (0.0)	2 (16.7)	0 (0.0)	0 (0.0)	1 (8.3)	12 (11.5)
Calcification	1 (16.7)	1(16.7)	3 (50.0)	0 (0.0)	0( 0.0)	0 (0.0)	1 (16.7)	6 (5.7)
Air bronchogram	3 (75.0)	1 (25.0)	0 (0.0)	0 (0.0)	0 (0.0)	0 (0.0)	0 (0.0)	4 (4.0)
Total								104 (100)

ADC : adenocarcinoma

EPC : epidermoid carcinoma

SCC : small cell carcinoma

HL : Hodgkin's lymphoma

NHL : non-Hodgkin's lymphoma

MT : metastasis

a Values expressed as n (%).

Benign lesions were more common in male patients (76%), tuberculosis being the most common in males and females (72%). There was a predominance of peripheral lesions (92%). Lesions with regular margins predominated (45.7%). Cavitation was the most common intralesional change (29.8%), followed by calcification (23.4%). Air bronchogram and cavitation were most common in tuberculosis patients (87.5% and 85.8%, respectively).

## Discussion

The present study evaluated the clinical application of CT and CT-PTNB in 113 patients with pulmonary nodules. The results of our study showed that CT and CT-PTNB were useful in distinguishing between malignant and benign lesions in patients with pulmonary nodules. Advanced age and smoking were significantly associated with malignancy. In patients with malignant pulmonary nodules, CT findings included larger diameter, spiculated margins, homogeneous attenuation, and necrosis. Adenocarcinoma was the most common malignant tumor, affecting mainly older patients. 

The mean age of the patients in the present study was 59.3 years, being similar to that found in the literature.^(^ 15 ^,^ 16 ^)^ In the present study, 23.0% of the patients with nodules and 54.8% of those with masses were found to have malignant lesions, the mean age of those patients ranging from 37.9 years (Hodgkin's lymphoma) to 65.6 years (epidermoid carcinoma). 

In patients under 40 years of age, the incidence of lung cancer is lower than 5%.^(^ 15 ^,^ 16 ^)^ This is due to the fact that advanced age increases the risk of lung cancer, which rarely occurs in individuals under 30 years of age.^(^ 15 ^,^ 17 ^)^ Lung cancer is currently a public health problem and is the leading cause of cancer death in males and females, the worldwide incidence of lung cancer increasing by 0.5% per year.^(^ 17 ^,^ 18 ^)^ In Brazil, lung cancer is the second leading cause of death in males and females.^(^ 19 ^)^ In the present study, 60.2% of all males and 39.8% of all females had lung cancer. This result is similar to those found in the literature.^(^ 19 ^-^ 21 ^)^

Regarding smoking, the proportion of malignant pulmonary nodules was higher in smokers than in nonsmokers (76.1% vs. 23.9%), malignant pulmonary nodules being more common in males (61.5%). These findings are consistent with the literature; however, the number of cases of malignancy in females is increasing because of smoking, lung cancer in females accounting for approximately half of all cases of lung cancer. ^(^ 15 ^,^ 17 ^,^ 18 ^)^ Smoking is the main risk factor for lung cancer, accounting for 80-90% of all cases.^(^ 15 ^,^ 17 ^,^ 21 ^)^

In the present study, CT scans revealed malignant lesions larger than 3 cm in diameter (lung masses) in 69% of the sample as a whole, a finding that suggests that most of the patients had advanced disease. This is probably due to the delayed onset of lung cancer symptoms and the difficulty in screening the population at risk.^(^ 22 ^)^ This result is consistent with the literature; the probability of malignancy is higher in individuals with lung masses (> 3 cm).^(^ 12 ^,^ 15 ^,^ 16 ^)^ Nevertheless, the results of the Early Lung Cancer Action Project^(^ 16 ^)^ showed that 8% of all nodules smaller than 1 cm in diameter were malignant. In the present study, malignant lesions ≤ 3 cm in diameter were detected in 23% of the patients. 

In the present study, approximately half of all malignant lesions were spiculated. In patients with pulmonary lesions, the presence of spicules is a predictor of malignancy in 90% of cases.^(^ 9 ^,^ 10 ^)^ In the present study, 28.7% of the lesions had irregular margins and 12.6% were bosselated. Although irregular margins and bosselated margins are suggestive of malignancy, they can also be found in benign lesions^(^ 9 ^,^ 10 ^,^ 23 ^)^; 25.7% of all benign lesions in the present study were found to have irregular margins, whereas 17.1% were found to have bosselated margins. 

Although homogeneous attenuation was the most common intralesional change in the patients with malignant nodules (being found in 51.9%), it cannot be used in order to distinguish between benign and malignant lesions, because other changes, such as necrosis, cavitation, and air bronchogram, are also indicative of malignancy,^(^ 12 ^)^ whereas calcification is the most common intralesional change in patients with benign lesions.^(^ 4 ^,^ 6 ^,^ 9 ^,^ 10 ^)^ In the present study, calcification was found in only 5% of all malignant lesions. 

We analyzed the histopathological reports and found that most (77.9%) of the pulmonary nodules were malignant, adenocarcinoma and epidermoid carcinoma being the most common tumors (38.0% and 14.1%, respectively). Adenocarcinoma is the most common tumor (in 30-50% of cases), followed by epidermoid carcinoma (in 30% of cases).^(^ 15 ^,^ 18 ^,^ 24 ^)^ Although the proportion of patients with epidermoid carcinoma in the present study was almost half that reported in the literature, this finding is related to intralesional changes, such as necrosis and cavitation, which were more common in those with that type of tumor, a finding that is consistent with the literature.^(^ 9 ^)^

More than 50% of all adenocarcinomas found in the present study were located in the peripheral region, a finding that is similar to that of another study.^(^ 9 ^)^ However, all epidermoid carcinomas in the present study were located in the peripheral region, and this is in disagreement with the results of a study showing that the central region is the most affected.^(^ 25 ^)^

Of all benign lesions found in the present study, those caused by tuberculosis were found to be the most common, a finding that is consistent with the literature showing that infectious granulomas are the most common cause of benign pulmonary nodules.^(^ 6 ^)^

Because of the characteristics of lung cancer progression, including late clinical symptoms associated with an absence of effective screening programs for the general population, lung cancer has become a serious clinical problem, helical CT being essential for detecting, characterizing, and biopsying such tumors. Lung cancer screening campaigns involving the use of multidetector CT and low radiation doses were found to reduce the risk of delayed diagnosis or lung cancer death in at-risk patients.^(^ 12 ^,^ 26 ^,^ 27 ^)^ However, lung cancer screening is not part of public health programs.^(^ 26 ^,^ 27 ^)^

In the present study, CT-PTNB contributed to the diagnosis of pulmonary nodules, avoiding unnecessary surgery or assisting in the treatment of malignant lung tumors. Therefore, according to a group of authors,^(^ 28 ^)^ pulmonary nodules require a multidisciplinary approach involving pulmonologists, thoracic surgeons, and radiologists. 
