# Supplementary material for: Clinical application of CT and CT-guided percutaneous transthoracic needle biopsy in patients with indeterminate pulmonary nodules
Source: J Bras Pneumol. 2014 Jul-Aug;40(4):380–8. doi: 10.1590/S1806-37132014000400005 (PMC4201168; doi:10.1590/S1806-37132014000400005)
Supplement: Translation

# Translation: Aplicação clínica da TC e biópsia transtorácica percutânea guiada por TC em pacientes com nódulos pulmonares indeterminados*

**Authors:** Luciana Vargas Cardoso, Arthur Soares Souza

**Affiliations:** Serviço de Radiologia, Hospital de Base de São José do Rio Preto, Faculdade de Medicina de São José do Rio Preto, São José do Rio Preto (SP) Brasil; Programa de Pós-Graduação, Faculdade de Medicina de São José do Rio Preto; e Diretor Administrativo. Instituto de Radiodiagnóstico Rio Preto-Ultra-X, São José do Rio Preto (SP) Brasil

**Keywords:** Nódulo pulmonar solitário, Tomografia, Biópsia guiada por imagem

## Abstract

**OBJETIVO:**

Investigar a aplicação clínica da TC e da biópsia transtorácica percutânea guiada por TC (BTP-TC) em pacientes com nódulos pulmonares indeterminados (NPIs).

**MÉTODOS:**

Foram estudados retrospectivamente 113 pacientes portadores de NPIs submetidos a TC e BTP-TC. Foram analisadas variáveis como sexo, idade ao diagnóstico, tabagismo, achados tomográficos e técnicas de BTP-TC. A análise dos dados foi efetuada por meio do teste t de Student para amostras independentes, teste do qui-quadrado e teste de comparação de duas proporções por aproximação normal.

**RESULTADOS:**

Dos 113 pacientes estudados, 68 (60,2%) eram do sexo masculino e 78 (69%) eram tabagistas. O diâmetro das lesões malignas variou de 2,6 a 10,0 cm. A maioria dos NPIs estava localizada na região periférica (85%). O resultado da biópsia foi maligno em 88 pacientes (77,8%) e benigno em 25 (22,2%). O adenocarcinoma foi o tumor maligno mais frequente, acometendo pacientes com idade mais avançada. O diâmetro dos NPIs foi significativamente maior nos pacientes com malignidade (p < 0,001). Houve uma associação significativa entre NPIs com contorno regular e lesões benignas (p = 0,022), enquanto os de tipo espiculado e bocelado foram mais frequentes em pacientes com lesões malignas (50,7% e 28,7%, respectivamente). Atenuação homogênea e necrose foram mais frequentes em pacientes com lesões malignas (51,9% e 26,9%, respectivamente).

**CONCLUSÕES:**

A TC e a BTP-TC foram úteis no diagnóstico diferencial entre lesões malignas e benignas nos pacientes com NPIs nesta amostra. Idade mais avançada e tabagismo associaram-se significativamente com malignidade. Houve associações de achados tomográficos (diâmetro maior, contorno espiculado, atenuação homogênea e necrose) com NPIs malignos.

## Introdução

Um dos maiores desafios na cirurgia torácica e na radiologia está relacionado à avaliação e à conduta no diagnóstico do paciente com nódulo pulmonar.^(1) ^O nódulo pulmonar é definido como uma opacidade focal visível em radiografia ou TC de tórax, de limites bem definidos, esférico, circundado por tecido pulmonar normal e com até 3 cm de diâmetro; quando maiores que 3 cm, recebem a denominação de massas.^(^ 2 ^)^

A investigação desse tipo de lesão é de extrema importância, pois é a manifestação mais frequente de câncer no pulmão, sendo um achado comum com o advento da TC de tórax. ^(3) ^Nos EUA, são detectados aproximadamente 150.000 nódulos pulmonares/ano.^(3,4) ^Trata-se de um achado radiológico no qual 60-70% dos casos são benignos e 30-40% são malignos.^(^ 4 ^)^

A abordagem do nódulo pulmonar envolve uma avaliação criteriosa do paciente, incluindo história clínica, exame físico, fatores de risco para malignidade e exames de diagnóstico por imagem.^(3,5) ^Dentre os métodos de diagnóstico por imagem que auxiliam na diferenciação entre nódulos pulmonares benignos e malignos, estão radiografia, TC, ressonância magnética, *positron emission tomography* (tomografia por emissão de pósitrons)/TC e biópsia transtorácica percutânea guiada por TC (BTP-TC).

A TC helicoidal é fundamental na diferenciação entre nódulos benignos e malignos, incluindo dados sobre tamanho, contorno do tumor, presença de calcificação interna e realce do nódulo por meio de contraste endovenoso. Além disso, permite maior acurácia na obtenção de fragmentos por biópsia.^(6,7) ^Na avaliação de pacientes com nódulo pulmonar, principalmente do sexo masculino, com idade acima de 50 anos, tabagistas e com história familiar de câncer ou fibrose pulmonar, a TC apresenta critérios como dimensão, localização, contorno, conteúdo, realce e tempo de duplicação.

A BTP-TC tem sido amplamente utilizada na investigação de nódulos e massas pulmonares. A coleta de material pode ser realizada por punção aspirativa por agulha fina (PAAF) ou por agulha grossa, essa última conhecida como *core biopsy*.^(8) ^Esse tipo de biópsia tem contribuído sobremaneira para o diagnóstico específico e precoce de nódulos pulmonares malignos, reduzindo as taxas de morbidade e mortalidade.^(^ 8 ^)^

O diagnóstico diferencial de nódulos pulmonares engloba diversas doenças e tumores. Nódulos benignos são representados principalmente por hamartoma, granuloma e linfonodo intrapulmonar.^(4) ^Granulomas infecciosos correspondem a 90% dos nódulos benignos, tendo como causas mais comuns tuberculose, histoplasmose e coccidioidomicose.^(4) ^Dentre os tumores malignos mais encontrados, destacam-se o adenocarcinoma e o carcinoma epidermoide.^(4)^

Diversos critérios tomográficos têm sido utilizados na diferenciação entre nódulos benignos e malignos. Nódulos de difícil delimitação, ausência de calcificação (central, laminada, difusa ou "em pipoca") ou gordura no interior da lesão, tempo de duplicação entre um mês e um ano aproximadamente e realce do nódulo maior que 15 UH por meio de contraste endovenoso em paciente acima da quarta década podem indicar doença maligna.^(7,9,10) ^Nódulos pequenos de limites nítidos, com calcificação concêntrica ou "em pipoca" em pacientes jovens podem sugerir lesão benigna.^(10) ^A estabilidade de crescimento da lesão por pelo menos dois anos também pode ser incluída como critério de benignidade.^(^ 11 ^)^

Considerando a relevância de pesquisas sobre a aplicação clínica da TC e BTP-TC em pacientes com nódulos pulmonares, justifica-se a realização do presente estudo, pois não foram encontrados trabalhos com esse enfoque na literatura nacional. Do ponto de vista clínico, a detecção precoce e a BTP-TC de lesões malignas, em alguns casos, podem evitar outros procedimentos invasivos, tais como biópsia via broncoscopia, videotoracoscopia ou até cirurgias desnecessárias. Com isso, a evolução do câncer pulmonar para estádios avançados pode ser evitada, melhorando a qualidade e a quantidade de vida do paciente.^(^ 3 ^,^ 12 ^)^

O objetivo do presente estudo foi investigar a aplicação clínica da TC e da BTP-TC em pacientes com nódulos pulmonares indeterminados, considerando-se características demográficas, tomográficas, aquelas referentes à BTP-TC e a correlação do diagnóstico histopatológico com essas variáveis.

## Métodos

De um total de 132 pacientes portadores de nódulos e massas pulmonares estudados no período entre junho de 2006 e maio de 2007, foram investigados retrospectivamente 113 pacientes (85,6%), independentemente de seu sexo, idade e raça, os quais foram submetidos à TC helicoidal e à BTP-TC. Esses procedimentos foram realizados no Serviço de Radiologia do Hospital de Base de São José do Rio Preto da Faculdade de Medicina de São José do Rio Preto, em São José do Rio Preto (SP). O estudo foi aprovado pelo comitê de ética em pesquisa da instituição (Protocolo nº 3682/2006).

Foram excluídos prontuários de 19 pacientes cujo laudo histopatológico apresentava resultados insatisfatórios ou inconclusivos devido à quantidade de material insuficiente.

Foram obtidos dos prontuários dos pacientes os seguintes dados: sexo; idade à época do diagnóstico; tabagismo; achados tomográficos, tais como diâmetro (nódulo ≤ 3 cm e massa > 3 cm),^(^ 2 ^)^ localização (central ou periférica), contorno (regular, irregular, espiculado ou bocelado) e alterações intralesionais (atenuação homogênea, necrose, cavitação, calcificação e broncograma aéreo); técnica utilizada na BTP-TC (PAAF, punção aspirativa com agulha grossa ou ambas); e complicações.

A avaliação dos achados tomográficos foi efetuada por dois radiologistas de forma independente e sem conhecimento prévio dos resultados histopatológicos.

Todos os exames foram realizados em aparelho de TC Tomoscan^(r)^ SR 4000 (Phillips Medical Systems, Eindhoven, Holanda). Foram realizados cortes tomográficos de 10 mm de espessura, desde os ápices até as bases pulmonares, em inspiração, utilizando um filtro de alta resolução para a reconstrução das imagens. Posteriormente, foi obtida uma nova sequência com aquisição helicoidal antes e após a injeção de contraste não iônico i.v., com cortes de 10 mm de espessura e *pitch* (razão entre deslocamento da mesa e espessura do corte) de 2 cm, utilizando-se um regime de 120 kVp com 150 mA.

Os pacientes submetidos à BTP-TC foram avaliados quanto ao estado geral, nível de consciência, reserva funcional pulmonar e parâmetros de coagulação. Todos foram informados quanto às complicações do procedimento e orientados em relação à respiração e em manter a apneia. O procedimento foi realizado sem a administração de contraste endovenoso, estando o paciente em decúbito dorsal ou ventral, visando possibilitar o acesso direto até a lesão, em pausa respiratória durante a inspiração. 

O protocolo do serviço de radiologia da instituição para a realização de BTP-TC incluiu cortes tomográficos de 5 a 10 mm de espessura, com *pitch* de 2 cm, utilizando-se um regime de 120 kVp com 150 mA, visando localizar a lesão, definir o nível apropriado para a introdução da agulha e medir a distância e a angulação da mesma. Foi feita a esterilização da região da punção com iodopovidona, cobertura com campos estéreis e administração de 10 mL de anestésico local (lidocaína a 2%). Foi realizada uma incisão pequena com bisturi (lâmina no. 14), por onde a agulha foi introduzida no tecido subcutâneo. Cortes tomográficos foram realizados para localizar a extremidade da agulha, sendo essa acoplada a uma pistola automática Bard Magnum^(r)^ (Manan Medical Products, Northbrook, IL, EUA).

As técnicas de BTP-TC utilizadas incluíram PAAF, biópsia *core* ou ambas as técnicas. Para a biópsia *core* foram utilizadas agulhas 18-20 G, enquanto para PAAF, essas eram 22-25 G. Após a biópsia, o paciente permaneceu monitorado por 2-3 h, incluindo a realização de cortes tomográficos a fim de detectar complicações.

Na análise de dados, foram utilizados cálculos de estatística descritiva e inferencial. Para a comparação de médias, foram aplicados o teste t de Student para amostras independentes (faixa etária e diâmetro do nódulo pulmonar em relação a lesões benignas ou malignas), o teste do qui-quadrado (sexo, localização da lesão, tabagismo e técnicas de BTP-TC em relação a lesões benignas ou malignas) e o teste de comparação de duas proporções com aproximação normal (contorno e alterações intralesionais em relação a lesões benignas ou malignas).^(^ 13 ^)^ O nível de significância adotado foi de p < 0,05. Todas as análises foram efetuadas por meio do programa Minitab versão 15 (Minitab Inc., State College, PA, EUA).^(^ 14 ^)^

## Resultados

Dos 113 pacientes estudados, 68 (60,2%) eram do sexo masculino e 45 (39,8%) eram do sexo feminino. A média de idade dos pacientes foi de 59,3 ± 12,6 anos e mediana de 61 anos (variação, 12-82 anos). Dentre os pacientes analisados, 78 (69%) eram fumantes e 35 (31%) eram não fumantes. Dos fumantes, 48 (61,5%) e 30 (38,5%) eram do sexo masculino e feminino, respectivamente.

O diâmetro das lesões pulmonares benignas variou de 1,8 a 6,5 cm e o das malignas variou de 2,6 a 10,0 cm. A relação entre nódulos e massas benignas ou malignas foi estatisticamente significativa (p = 0,003), sendo mais frequente a malignidade tanto para nódulos (23,0%) como para massas pulmonares (54,8%). Nódulos pulmonares foram mais frequentemente detectados na região periférica pulmonar (85% dos casos) e menos frequentemente na região central (15% dos casos). Houve um predomínio de tumores malignos nos lobos superiores, em 67 pacientes (76%). Nos 113 pacientes estudados, foi encontrado um total de 185 contornos de nódulos pulmonares, sendo o de tipo espiculado o mais frequente (49,7%), seguido do tipo bocelado (26,5%), irregular (12,4%) e regular (11,4%; Figura 1). Os achados tomográficos mostraram um total de 151 alterações intralesionais, sendo o aspecto homogêneo o mais frequente (42,4%), seguido de necrose (21,2%), cavitação (17,2%), calcificação (11,2%) e broncograma aéreo (8,0%; Figura 2).

**Figura 1:** Cortes de TC helicoidal mostrando contornos de massa pulmonar irregular (em A; paciente masculino, 77 anos) e dos tipos bocelado (em B; paciente masculino, 30 anos), espiculado (em C; paciente masculino, 64 anos) e regular (em D; paciente masculino, 36 anos).

**Figura 2:** Cortes de TC helicoidal mostrando alterações intralesionais com aspecto homogêneo (em A; paciente masculino, 49 anos), necrose (em B; paciente masculino, 36 anos), calcificação (em C; paciente masculino, 56 anos) e broncograma aéreo (em D; paciente masculino, 77 anos).

A técnica de PAAF foi realizada em 71 pacientes, enquanto a por agulha grossa foi realizada em 81 pacientes, sendo ambas as técnicas utilizadas em 39 pacientes. O pneumotórax foi a única complicação decorrente da BTP-TC, sendo observada em 37 pacientes (32,7%). Com relação ao diagnóstico histopatológico, dos 113 pacientes que realizaram biópsia, o resultado foi maligno e benigno, respectivamente, em 88 (77,8%) e em 25 (22,2%). 

O diagnóstico histopatológico de pacientes com nódulos pulmonares em relação à faixa etária e ao diâmetro mostrou que o adenocarcinoma foi o tipo mais frequente (48,9%), acometendo pacientes mais idosos (média de idade = 65,6 ± 9,1 anos), e que o tamanho das lesões malignas variou de 2,4 a 10,0 cm, enquanto o das lesões benignas variou de 1,8 a 6,5 cm. 

Considerando-se a faixa etária, houve uma diferença significativa entre lesões benignas e malignas (p = 0,034), tendo idade mais avançada os pacientes com lesão maligna; quanto ao diâmetro, a diferença foi significativa entre as lesões benignas e malignas (p < 0,001), sendo esse maior nos pacientes com resultado histopatológico maligno (Tabela 1).

**Tabela 1:** Idade dos pacientes (N = 113) e diâmetro dos nódulos pulmonares em função do diagnóstico histopatológico das lesões na amostra estudada.

Variáveis	Diagnóstico	n	Média ± dp	Mediana (variação)	p*
Idade	Maligno	88	60,7 ± 12,1	63 (30-80)	0,034
	Benigno	25	54,4 ± 12,9	60 (12-82)	
Diâmetro	Maligno	88	5,3 ± 1,9	5,0 (2,4-10,0)	< 0,001
	Benigno	25	3,7 ± 1,3	4,0 (1,8-6,5)	

* Teste t de Student para amostras independentes.

Na comparação do diagnóstico histopatológico em relação ao sexo e aos achados tomográficos (Tabela 2), observa-se que sexo e localização de nódulo pulmonar não apresentaram associações significativas com o diagnóstico histopatológico (p = 0,067 e p = 0,264, respectivamente). Com relação ao contorno do nódulo pulmonar, o tipo regular mostrou uma associação significativa com o diagnostico histopatológico (p = 0,022), sendo mais frequente em lesões benignas. Os tipos espiculado e bocelado foram mais frequentes em pacientes com lesões malignas (50,7% e 28,7%, respectivamente). Houve associações significativas de todas as alterações intralesionais com o diagnóstico histopatológico. Atenuação homogênea e necrose foram mais frequentes em pacientes com lesões malignas (51,9% e 26,9%, respectivamente), enquanto cavitação, calcificação e broncograma aéreo foram mais encontradas naqueles com lesões benignas (29,8%, 23,4% e 17,0%, respectivamente). Nos cálculos referentes aos testes de comparação de proporções (Tabela 2), os achados tomográficos referentes a contorno lesional e alterações intralesionais foram analisados considerando-se que um mesmo paciente poderia ter apresentado mais de um tipo de contorno lesional e/ou de alteração intralesional.

**Tabela 2:** Sexo e achados tomográficos dos pacientes (N = 113) em função do diagnóstico histopatológico das lesões na amostra estudada.a

Parâmetros		Diagnóstico		Total	p
		Maligno	Benigno		
Sexo	Feminino	39 (44,3)	06 (24,0)	45 (39,8)	0,067*
	Masculino	49 (55,7)	19 (76,0)	68 (60,2)	
	Total	88 (100,0)	25 (100,0)	113 (100,0)	
Localização	Central	15 (17,0)	02 (8,0)	17 (15,0)	0,264*
	Periférica	73 (83,0)	23 (92,0)	96 (85,0)	
	Total	88 (100,0)	25 (100,0)	113 (100,0)	
Contorno lesional	Regular	12 (8,0)	16 (45,7)	28 (15,1)	0,022**
	Espiculado	76 (50,7)	09 (25,7)	85 (45,9)	0,597**
	Bocelado	43 (28,7)	06 (17,1)	49 (26,5)	0,118**
	Irregular	19 (12,6)	4 (11,4)	23 (12,4)	0,837**
	Total	150 (100,0)	35 (100,0)	185 (100,0)	
Alterações intralesionais	Atenuação homogênea	54 (51,9)	10 (21,3)	64 (42,4)	0,001**
	Necrose	28 (26,9)	04 (8,5)	32 (21,2)	0,007**
	Cavitação	12 (11,5)	14 (29,8)	26 (17,2)	0,004**
	Calcificação	06 (5,7)	11 (23,4)	17 (11,2)	0,003**
	Broncograma aéreo	04 (4,0)	08 (17,0)	12 (8,0)	0,015**
	Total	104 (100,0)	47 (100,0)	151 (100,0)	

a Valores expressos em n (%)

* Teste do qui-quadrado

** Teste de comparação de duas proporções por aproximação normal.

Houve uma associação significativa entre lesões malignas e tabagismo (p = 0,002), sendo a maioria dos pacientes constituída por tabagistas (n = 78), dos quais 76,1% apresentaram lesões malignas.

Não houve associação significativa entre diagnóstico histopatológico e técnicas de BTP-TC (p = 0,778). A distribuição percentual de diagnóstico maligno obtida pelas técnicas *core biopsy*, PAAF ou ambas foi próxima, isto é, 29,2%, 23,0% e 25,6%, respectivamente.

A distribuição percentual de lesões malignas conforme sexo e achados tomográficos encontra-se na Tabela 3. Essas lesões foram mais frequentes em pacientes do sexo masculino (55,7%). O adenocarcinoma foi o tipo mais frequente em ambos os sexos (48,9%). Com relação à localização, a mais frequente foi a periférica (82,9%). O adenocarcinoma foi o tumor mais comum na região periférica (56,2%). Quanto ao contorno, aproximadamente metade dos casos foi do tipo espiculado (50,7%). Em pacientes com adenocarcinoma, os contornos mais frequentes foram irregular (57,9%), espiculado (51,3%) e bocelado (44,2%). Dentre as alterações intralesionais, atenuação homogênea foi a mais comum (51,9%), seguida de necrose (26,9%). A atenuação homogênea foi mais detectada em pacientes com adenocarcinoma e carcinoma epidermoide (38,9% e 24,1%, respectivamente). A cavitação foi mais frequente em casos de carcinoma epidermoide (66,7%). 

**Tabela 3:** Tipos de lesões malignas encontradas em função do sexo e dos achados tomográficos dos pacientes estudados (N = 113).a

Parâmetros	Lesões malignas							
	ADC	CEP	LH	CPC	LNH	MT	Outras	Total
Sexo								
Feminino	15 (38,5)	11 (28,2)	5 (12,8)	0 (0,0)	2 (5,1)	1 (2,6)	5 (12,8)	39 (44,3)
Masculino	28 (57,1)	5 (10,2)	3 (6,1)	6 (12,2)	2 (4,1)	3 (6,1)	2 (4,1)	49 (55,7)
Total								88 (100)
Localização								
Central	2 (13,3)	0 (0,0)	8 (53,3)	2 (13,3)	1 (6,7)	0 (0,0)	2 (13,3)	15 (17,0)
Periférica	41 (56,2)	16 (21,9)	0 (0,0)	4 (5,6)	3 (4,1)	4 (5,6)	5 (6,8)	73 (83,0)
Total								88 (100)
Contorno								
Regular	4 (33,3)	4 (33,3)	1 (8,3)	0 (0,0)	0 (0,0)	1 (8,3)	2 (16,7)	12 (8,0)
Espiculado	39 (51,3)	12 (15,8)	7 (9,2)	6 (7,9)	4 (5,3)	3 (3,9)	5 (6,6)	76 (50,7)
Bocelado	19 (44,2)	6 (14,0)	7 (16,3)	3 (7,0)	1 (2,3)	3 (7,0)	4 (9,3)	43 (28,7)
Irregular	11 (57,9)	3 (15,8)	0 (0,0)	3 (15,8)	0 (0,0)	1 (5,3)	1 (5,3)	19 (12,6)
Total								150 (100)
Alteração intralesional								
Atenuação homogênea	21 (38,9)	13 (24,1)	7 (13,0)	3 (5,6)	1 (1,9)	4 (7,4)	5 (9,3)	54 (51,9)
Necrose	17 (60,7)	3 (10,7)	1 (3,6)	2 (7,1)	3 (10,7)	0 (0,0)	2 (7,1)	28 (26,9)
Cavitação	1 (8,3)	8 (66,7)	0 (0,0)	2 (16,7)	0 (0,0)	0 (0,0)	1 (8,3)	12 (11,5)
Calcificação	1 (16,7)	1(16,7)	3 (50,0)	0 (0,0)	0( 0,0)	0 (0,0)	1 (16,7)	6 (5,7)
Broncograma aéreo	3 (75,0)	1 (25,0)	0 (0,0)	0 (0,0)	0 (0,0)	0 (0,0)	0 (0,0)	4 (4,0)
Total								104 (100)

ADC : adenocarcinoma

CEP : carcinoma epidermoide

CPC : carcinoma de pequenas células

LH : linfoma de Hodgkin

LNH : linfoma não Hodgkin

MT : metástase

a Valores expressos em n (%).

Lesões benignas foram mais frequentes em pacientes do sexo masculino (76%), sendo a tuberculose mais comum em ambos os sexos (72%). Houve um predomínio da localização periférica (92%). O contorno das lesões do tipo regular foi o mais frequente (45,7%). A cavitação foi a alteração intralesional mais frequente (29,8%), seguida de calcificação (23,4%). Pacientes com tuberculose apresentaram mais frequentemente broncograma aéreo (87,5%) e cavitação (85,8%).

## Discussão

O presente estudo avaliou a aplicação clínica da TC e a BTP-TC em 113 pacientes com nódulos pulmonares. Os resultados da presente pesquisa mostraram que a TC e a BTP-TC em pacientes com nódulos pulmonares foram úteis no diagnóstico diferencial entre lesões malignas e benignas. Idade mais avançada e tabagismo apresentaram associações significativas com malignidade. Dentre os achados tomográficos associados com nódulos pulmonares malignos, destacam-se o diâmetro maior, o contorno do tipo espiculado e as alterações intralesionais (atenuação homogênea e necrose). O adenocarcinoma foi o tumor maligno mais frequente, acometendo principalmente pacientes mais idosos.

A média de idade dos pacientes estudados foi de 59,3 anos, semelhante à encontrada na literatura.^(^ 15 ^,^ 16 ^)^ Na presente série, 23,0% dos pacientes com nódulos e 54,8% dos pacientes com massas apresentaram lesões malignas, com média de idade variando de 37,9 anos (linfoma de Hodgkin) a 65,6 anos (carcinoma epidermoide). 

Em pacientes com menos de 40 anos, a incidência de câncer de pulmão é menor que 5%.^(^ 15 ^,^ 16 ^)^ Isso é justificado, pois a idade avançada aumenta o risco dessa neoplasia, que é rara antes dos 30 anos.^(^ 15 ^,^ 17 ^)^ Atualmente, o câncer de pulmão representa um problema de saúde pública, sendo a principal causa de morte por neoplasia em homens e mulheres, com um aumento de 0,5% ao ano em sua incidência mundial. ^(^ 17 ^,^ 18 ^)^ No Brasil, o câncer de pulmão ocupa o segundo lugar em causa de morte em homens e mulheres. ^(^ 19 ^)^ No presente estudo, 60,2% dos homens e 39,8% das mulheres apresentaram essa neoplasia. Esse resultado foi similar aos encontrados na literatura.^(^ 19 ^-^ 21 ^)^

Com relação ao tabagismo, a quantidade total de nódulos pulmonares malignos em pacientes tabagistas (76,1%) foi superior à encontrada em pacientes não tabagistas (23,9%), sendo mais frequente no sexo masculino (61,5%). Esses achados são concordantes com aqueles na literatura; porém, o número de casos em mulheres está aumentando devido ao tabagismo, representando aproximadamente metade dos casos de câncer pulmonar.^(^ 15 ^,^ 17 ^,^ 18 ^)^ Vale ressaltar que o tabagismo é o principal fator de risco para o desenvolvimento do câncer de pulmão, sendo responsável por 80-90% dos casos.^(^ 15 ^,^ 17 ^,^ 21 ^)^

Na presente pesquisa, a TC de 69% dos pacientes avaliados mostrou lesões malignas com diâmetro maior que 3 cm (massas pulmonares), sugerindo que a maioria dos pacientes já estava em estádio avançado. Isso provavelmente pode estar relacionado à demora no aparecimento de sintomas relacionados ao câncer de pulmão e às dificuldades de rastreamento na população de risco.^(^ 22 ^)^ Esse resultado está de acordo com os encontrados na literatura, pois a probabilidade de malignidade é maior em casos de massas pulmonares (> 3 cm).^(12,15,16) ^Por outro lado, segundo resultados obtidos no estudo *Early Lung Cancer Action Project*,^(^ 16 ^)^ 8% dos nódulos com diâmetro menor que 1 cm eram malignos. No presente estudo, foram detectadas lesões malignas com diâmetro ≤ 3 cm, em 23% dos pacientes. 

No presente estudo, aproximadamente metade das lesões malignas apresentou contorno do tipo espiculado. A presença de espículas em lesões pulmonares é um fator preditivo de malignidade em 90% dos casos.^(^ 9 ^,^ 10 ^)^ Na presente série, foram identificados também contornos do tipo irregular e bocelado, em 28,7% e 12,6% das lesões, respectivamente. Apesar de indicarem malignidade, os contornos do tipo irregular e bocelado podem ocorrer em lesões benignas,^(^ 9 ^,^ 10 ^,^ 23 ^)^ como encontrado em nossos resultados (em 25,7% e 17,1% das lesões, respectivamente).

Analisando as alterações intralesionais em nódulos malignos, apesar de a atenuação homogênea ter sido mais frequente (51,9%), essa não pode ser utilizada como critério na diferenciação entre lesões benignas e malignas, pois outras alterações, tais como necrose, cavitação e broncograma aéreo, são também indicadoras de malignidade,^(^ 12 ^)^ enquanto a calcificação é a mais comum em lesões benignas.^(^ 4 ^,^ 6 ^,^ 9 ^,^ 10 ^)^ No presente estudo, a calcificação esteve presente em apenas 5% das lesões malignas.

Após a análise dos laudos histopatológicos, a maioria dos nódulos pulmonares foi maligna (77,9%), sendo o adenocarcinoma e o carcinoma epidermoide os mais frequentes (38,0% e 14,1%, respectivamente). O adenocarcinoma é o tumor mais comum (em 30-50% dos casos), seguido do carcinoma epidermoide (em 30% dos casos). ^(^ 15 ^,^ 18 ^,^ 24 ^)^ Embora a quantidade de pacientes com carcinoma epidermoide tenha sido quase a metade do referido na literatura, esse achado está relacionado com alterações intralesionais, como necrose e cavitação, que ocorreram com maior frequência nesse tipo de tumor, corroborando dados da literatura.^(^ 9 ^)^

Em mais de 50% dos casos de adenocarcinoma, a localização foi periférica, sendo similar ao encontrado em um estudo.^(^ 9 ^)^ Por outro lado, a localização do carcinoma epidermoide foi periférica em todos os nódulos avaliados, o que difere dos resultados em um estudo, que mostra a região central como a mais acometida.^(^ 25 ^)^

No presente estudo, dentre as lesões benignas detectadas, a mais comum foi devida à tuberculose, estando de acordo com a literatura, cujos dados indicam que os granulomas infecciosos são a causa mais comum de nódulos pulmonares benignos.^(^ 6 ^)^

Devido a características evolutivas do câncer de pulmão, como sintomas clínicos tardios associados à ausência de programas efetivos de rastreamento na população geral, essa neoplasia tornou-se um problema clínico grave, sendo a TC helicoidal fundamental na detecção, caracterização e biópsia desse tumor. Campanhas de rastreamento por meio de TC com multidetectores e doses baixas de radiação comprovaram a diminuição no risco de diagnóstico tardio ou de morte por câncer de pulmão em pacientes de risco.^(^ 12 ^,^ 26 ^,^ 27 ^)^ Porém, não há um método de rastreamento dessa neoplasia em programas de saúde pública.^(^ 26 ^,^ 27 ^)^

No presente estudo, a BTP-TC contribuiu no diagnóstico de nódulos pulmonares, evitando cirurgias desnecessárias ou auxiliando no tratamento específico de tumores pulmonares malignos. Portanto, a abordagem de nódulos pulmonares, conforme um grupo de autores,^(^ 28 ^)^ deve ser multidisciplinar, envolvendo profissionais das áreas de pneumologia, cirurgia torácica e radiologia.
